# Molecular origin of sliding friction and flash heating in rock and heterogeneous materials

**DOI:** 10.1038/s41598-020-79383-y

**Published:** 2020-12-17

**Authors:** Nariman Piroozan, Muhammad Sahimi

**Affiliations:** Mork Family of Department of Chemical Engineering and Materials Science, University of Southern California, Los Angeles, CA 90089-1211 USA

**Keywords:** Mathematics and computing, Engineering, Chemical engineering

## Abstract

It is generally believed that earthquakes occur when faults weaken with increasing slip rates. An important factor contributing to this phenomenon is the faults’ dynamic friction, which may be reduced during earthquakes with high slip rates, a process known as slip-rate weakening. It has been hypothesized that the weakening phenomenon during fault slip may be activated by thermal pressurization of pores’ fluid and *flash heating*, a microscopic phenomenon in which heat is generated at asperity contacts due to high shear slip rates. Due to low thermal conductivity of rock, the heat generated at the contact points or surfaces cannot diffuse fast enough, thus concentrating at the contacts, increasing the local contact temperature, and reducing its frictional shear strength. We report the results of what we believe to be the first *molecular scale* study of the decay of the interfacial friction force in rock, observed in experiemntal studies and attributed to flash heating. The magnitude of the reduction in the shear stress and the local friction coefficients have been computed over a wide range of shear velocities *V*. The molecular simulations indicate that as the interfacial temperature increases, bonds between the atoms begin to break, giving rise to molecular-scale fracture that eventually produces the flash heating effect. The frequency of flash heating events increases with increasing sliding velocity, leaving increasingly shorter times for the material to relax, hence contributing to the increased interfacial temperature. If the material is thin, the heat quickly diffuses away from the interface, resulting in sharp decrease in the temperature immediately after flash heating. The rate of heat transfer is reduced significantly with increasing thickness, keeping most of the heat close to the interface and producing weakened material. The weakening behavior is demonstrated by computing the stress–strain diagram. For small strain rates there the frictional stress is essentially independent of the materials’ thickness. As the strain rate increases, however, the dependence becomes stronger. Specifically, the stress–strain diagrams at lower velocities *V* manifest a pronounced strength decrease over small distances, whereas they exhibit progressive increase in the shear stress at higher *V*, which is reminiscent of a transition from ductile behavior at high velocities to brittle response at low velocities.

## Introduction

Sliding friction between contacting surfaces has been an enduring scientific problem for many years^1^. While one aspect of the problem’s significance has been its role in the fabrication of engineering surfaces capable of operating at high velocities, another equally important aspect is its utmost importance to understanding the nature of sliding friction in rock at subseismic slip rates^2,3^, the focus of this paper. It is generally believed that earthquakes occur as a result of faults’ weakening over time^4,5^, but the thermal and mechanical reasons for the weakening is still not well understood, particularly in the light of the fact that studies of the phenomenon have been restricted mostly to either experimental investigations or phenomenological models. To our knowledge, the molecular origin of sliding friction in rock and its consequences have never been studied.

As is well known, there exist two primary types of kinetic friction between opposing surfaces. One is wet or lubricated sliding friction, which is described by the Stribeck curve^6,7^, according to which there is a nonlinear relationship between lubricated interfaces and contact load. While wet sliding friction is well understood, the same cannot be claimed about the second type of kinetic friction, namely, sliding friction between dry surfaces. This type of sliding friction is generally described via Coulomb’s law^8^ according to which the magnitude of the kinetic friction force $F_k$ is given by, $F_k=\mu F_n$, where $\mu$ is the friction coefficient, and $F_n$ is the applied normal force $F_n$ between the two surfaces. Coulomb’s law implies that $F_k$ is independent of the sliding velocity. Recent theoretical and computational work^9^ has, however, shown that $F_k$ is in fact a function of the velocity.

When two rock surfaces, which are typically rough, are brought into contact, they touch over only a small fraction of their nominal contact area. Sliding generates heat, which should increase the temperature. If the slip rate between the two surfaces is small, most of the generated heat at the contacting points diffuses away and, therefore, temperature will rise only slightly, with its effect on the contact strength being negligible. On the other hand, due to the low thermal conductivity of rock (see below), the generated heat at high slip rates cannot diffuse away fast enough and, therefore, it increases the contact temperature and decreases the contact strength. Thus, large contact stresses and sliding velocities induce intense dynamic heating of the contacts, resulting in low shear strengths and low macroscopic friction. Indeed, experimental studies have indicated^10^ that under such conditions there is a precipitous decrease in the friction coefficient as a function of the sliding velocity. This phenomenon, referred to as *flash heating*^11–18^, has been known since the 1950s^19–21^, and occurs even while the average temperature of the slip surface is hardly above the initial temperature. Given the low thermal conductivity of rock, there is also a significant risk of thermal fatigue over time^22,23^.

The purpose of this paper is to study, at molecular scale, sliding friction at high velocities between two typical rock surfaces and the resulting flash heating. Although our goal in this paper is to understand flash heating in rock at molecular scale, the approach and results have much wider applications, ranging from the abrasive wear on carbon-ceramic disk brakes used in Formula One race cars^24^, to many others. As one of the most abundant minerals in Earth’s continental crust^25^, which also has a high melting point, quartz—SiO$_2$—is an indeal rock-type model material for our study. Sandstones, for example, are mostly—up to 90%—made of quartz. Experimental studies of sliding friction between quartz surfaces indicated that the friction coefficient decreases precipitously when the sliding velocity increases to high values^13,15^.

We use molecular dynamics (MD) simulations that make it possible to incorporate the precise chemical composition of the material under inspection, and study the effect of both thermal and mechanical properties on high-velocity sliding friction and flash heating. Three atomistic systems of different thicknesses are generated, each of which contain a pair of equal-size slabs. The dimensions of the smallest slab, referred to as Structure A, were $86.8 \times 50.1\times 32.8\;\AA ^3$ in the *xyz* directions. The dimensions of the second slab, Structure B, was $86.8\times 50.1\times 82.0\;\AA ^3$, while the thickest slab, Structure C, was built with dimensions of $86.8\times 50.1\times 131.3\;\AA ^3$. The three structures had the same interfacial contact area, but different thicknesses, hence allowing us to understand the effect of not only the sliding velocity, but also the thickness on kinetic friction and flash heating, and the resulting temperature profiles.

## Results

**Figure 1:**
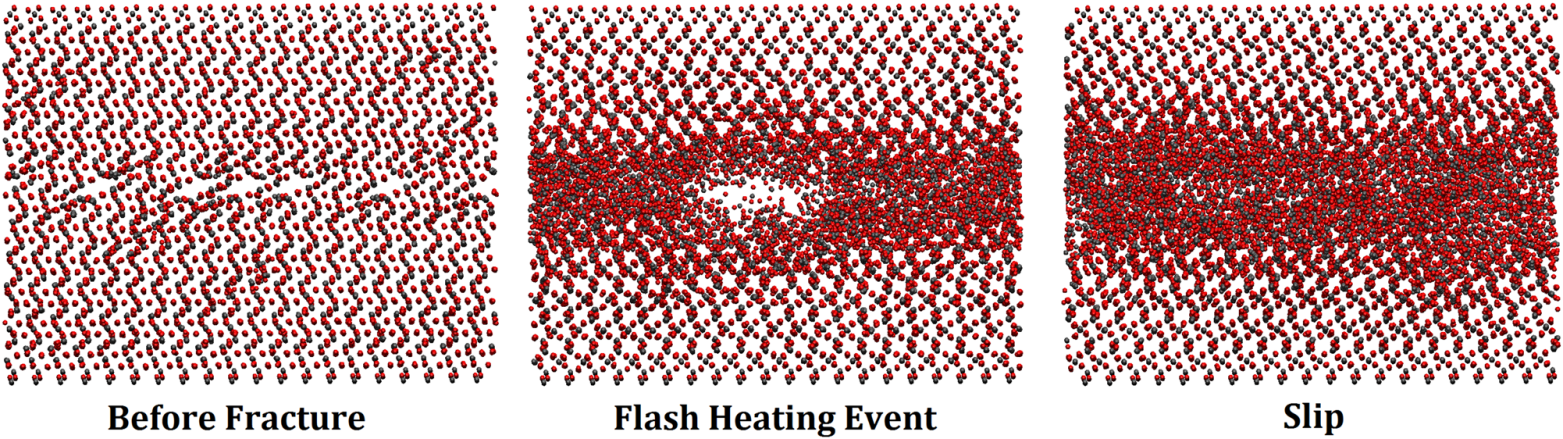
Dynamic evolution of Flash heating event for Structure A at a velocity of 0.3 m/s.

We first study flash heating and material weakening, since sliding between the two slabs produces heat, which is most acute near the interface between the slabs. Figure 1 presents the evolution of the structural configuration of Structure A at a sliding velocity of 0.3 m/s. Just prior to the onset of interfacial fracture, there is a large buildup of energy that manifests itself in the form of an interfacial cavity during the fracture event. When the two layers are in contact, the interfacial region becomes amorphous. However, because the material is exposed to very high pressure, the cavity disappears quickly. This phenomenon is the root cause of flash heating at the interface. Its effect and contribution to the weakening of the material is quite fascinating, as described below.

**Figure 2:**
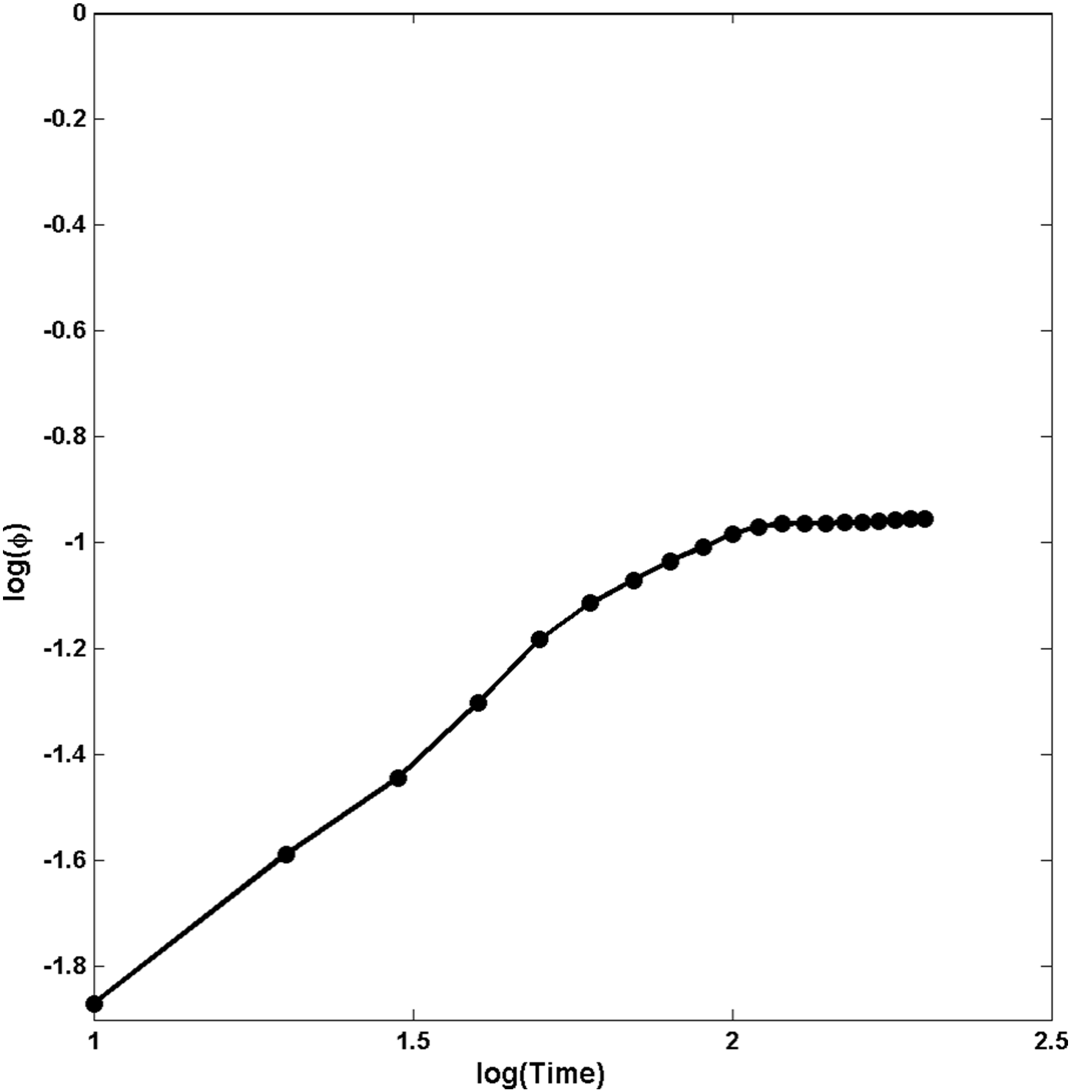
The dynamic growth of the normalized thickness $\phi$ of the amorphous region in Structure C. The slope of the straight line is $\alpha \approx 0.9$.

How fast does the amorphous region grow with the time? We carried out MD simulations for 5 realizations of Structure C (with the highest thickness) and computed the time-dependence of the thickness of the amorphous region. Figure 2 presents the logarithmic plot of the dynamic evolution of the average thickness $\phi$ of the region, normalized by the total thickness of the structure, for a velocity of $V=1.5$ cm/s. The results indicate that,

1 $$\begin{aligned} \phi \propto t^\alpha \;, \end{aligned}$$

where, $\alpha \approx 0.9\pm 0.02$. Eventually, of course, the thickness of the amorphous region saturates, as there is not enough heat to propagate deeper and break the bonds. Thus, the growth appears to be superdiffusive, faster than diffusive one for which $\alpha =1/2$. We found similar results for other velocities.

**Figure 3:**
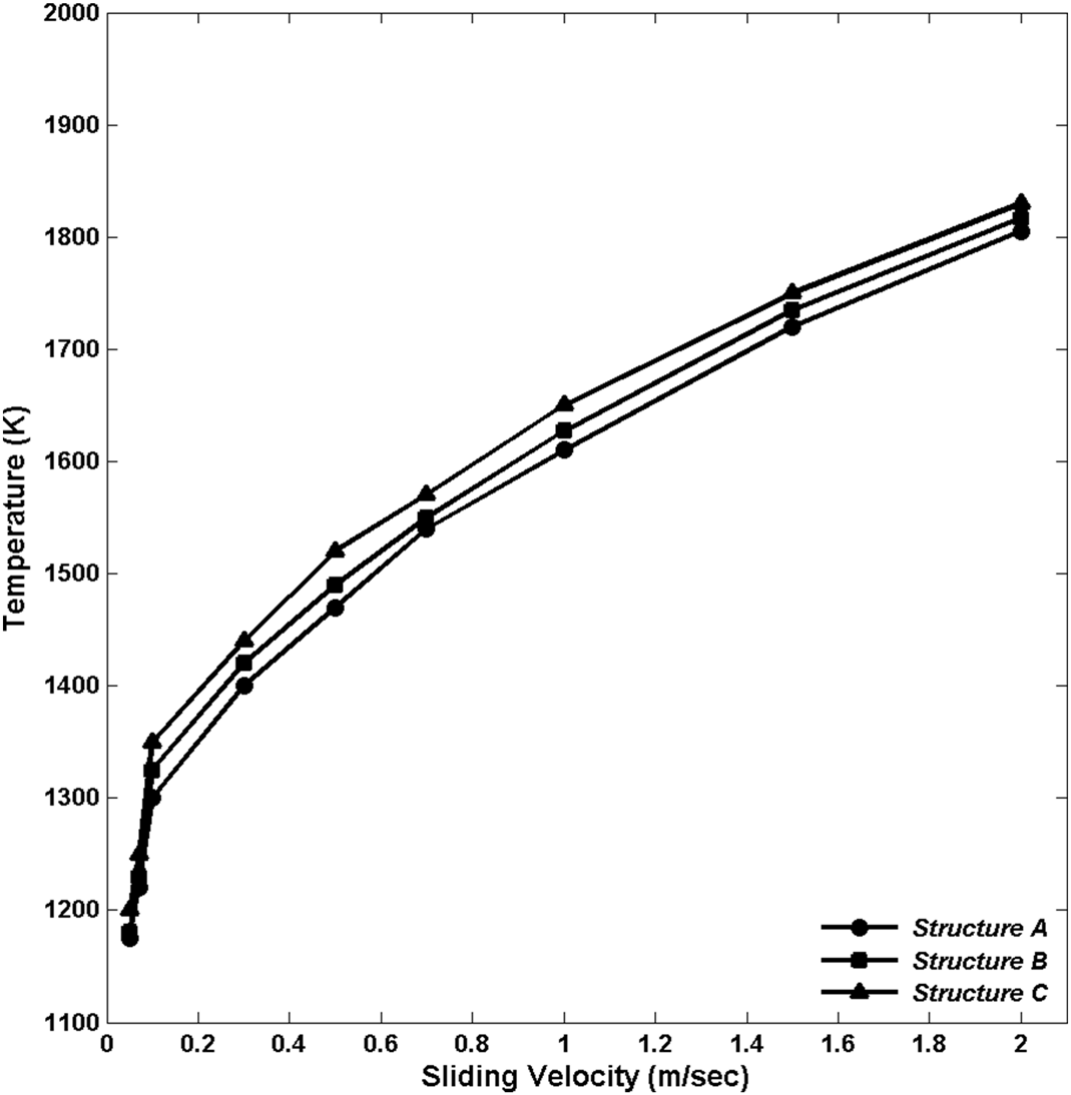
Maximum interfacial temperature during flash heating events across the entire spectrum of simulated velocities and Structures.

In Fig. 3 we show the maximum temperature at the interface for the three structures over the course of the simulation for all the velocities. As the sliding velocity increases, the interfacial temperature rises dramatically until a velocity of 0.3 m/s is reached, beyond which there is gradual tempering of the temperature increase. It should be noted that at no time during the simulations did the sustained interfacial temperature exceed the melting point of quartz. Furthermore, Fig. 3 does not represent the sustained interfacial temperature, but rather its maximum value, representing the flash heating event.

**Figure 4:**
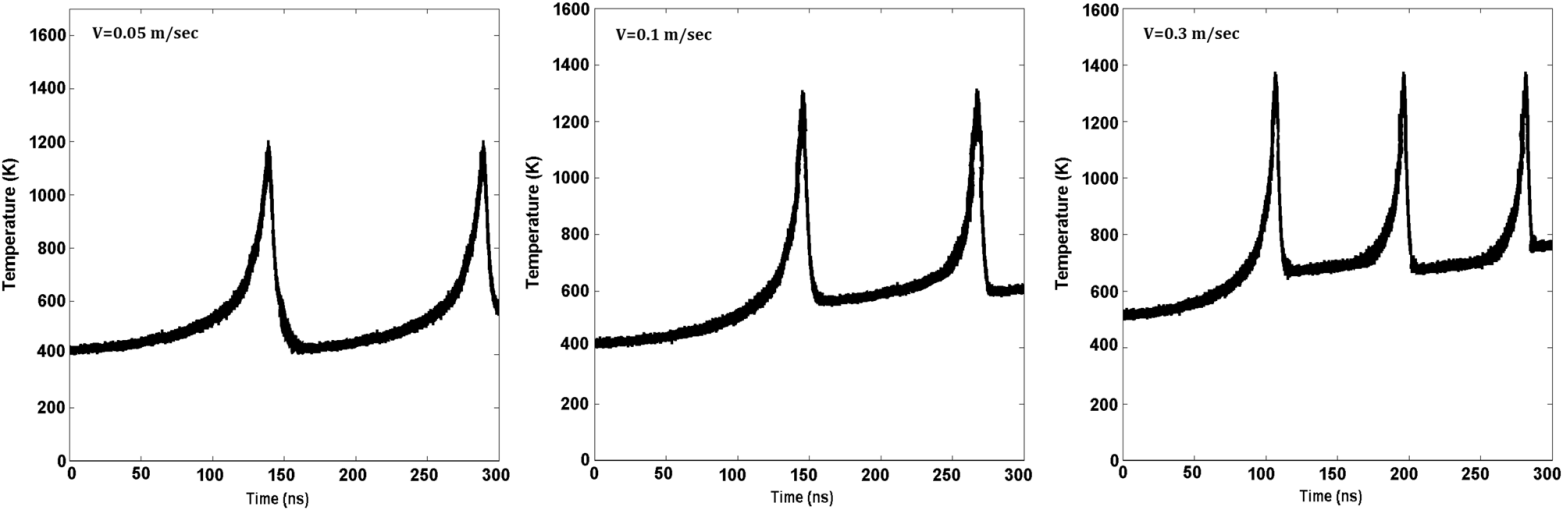
Temperature profiles during flash heating events in Structure C for several velocities.

Figure 4 demonstrates the flash heating event during dynamic evolution of the temperature at the interface in Structure C as a function of the sliding velocity. The fascinating aspect of the results is not only the increase in the frequency of sharp rises in the temperature as the sliding velocity increases, but also the increase in the interfacial temperature between pairs of the sharp increase. Moreover, after the first flash event due to the lowest sliding velocity, the temperature at the interface reverts to its original level prior to the event’s occurrence, hence indicating that the rate of heat transfer through the material is sufficiently high so as to allow this to occur. As the sliding velocity increases, however, the temperature of the interface after each flash event increases because there is not enough time for the heat to diffuse away before the next event.

Note that, due to the flash heating events, one has interfacial cavities due to the sudden spikes in the temperature that reduce the contact area (see Fig. 1). If the cavities persist for a significant amount of time, it would make it difficult to accurately compute the friction coefficient. But the event is exceptionally transitory, and does not impact the nature of the results. Note also that the “melting” that the MD simulations indicate is not sustained. while it is true, as Fig. 4 indicates, that instantaneous melting is present, the temperature quickly drops after the slip event, and the material seems to heal. In addition, no melting occurs in the bulk. We also point out that, in principle, interface shearing at high pressures or high speeds may cause surface grinding or breakage that may reduce friction. The simulation did not, however, provide any evidence for this phenomenon under the conditions that we studied.

**Figure 5:**
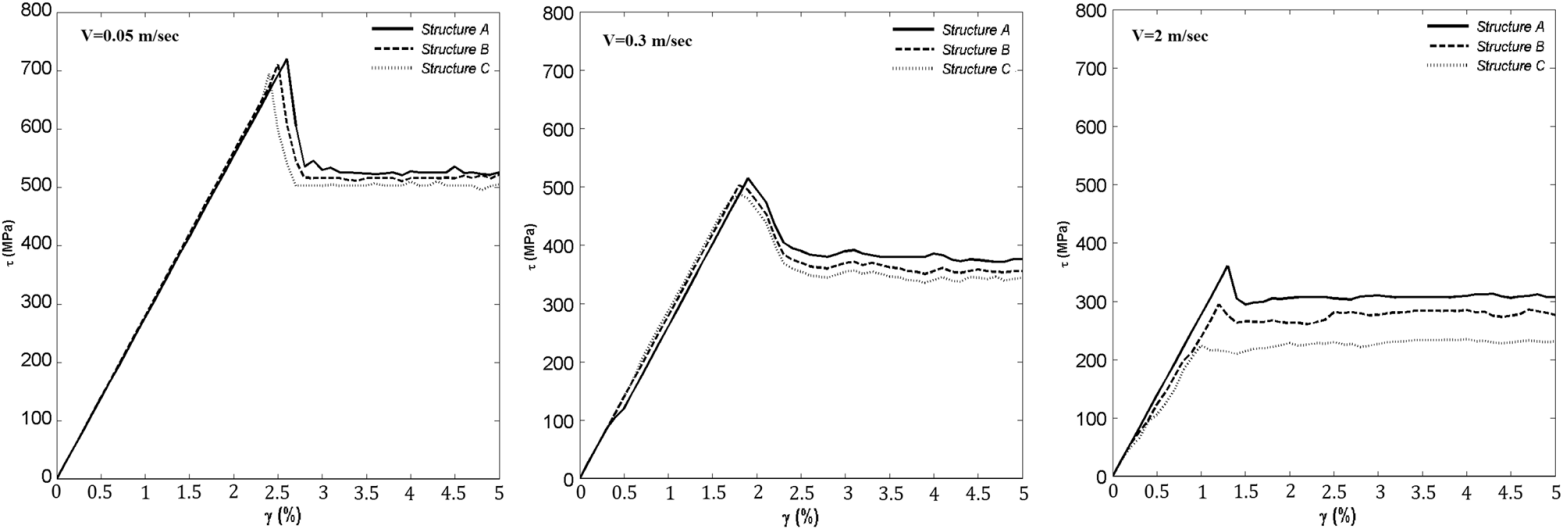
Shear stress–shear strain diagrams for the three structures for several velocities.

To better understand this, we present in Fig. 5 the shear stress–shear strain diagrams for the three structures and three sliding velocities, where the shear stress is simply the ratio of the sum of all the forces exerted on the top layer (TL; see “Methods” section below) and the surface area *A* in the *xy* plane. The friction force $F_x$ was also computed based on the potential energy *U*(*x*), namely, through the relation, $F_x=-(1/A)dU/dx$. As $F_x$ represents the total lateral atomic force on the TL, we also computed the friction force at each successive MD time step. Similarly, in order to compute the shear strain, the positions of the centers of mass of both the TL and bottom layer (BL) at each MD time step were determined, based on which the shearing distance was computed.

Figure 5 reveals the change in the behavior of the material as a function of both the thickness and the sliding velocity. With increasing velocity, a number of changes takes place. At a sliding velocity of 0.05 m/s, the shear stress is very large, indicating a significant friction force at the interface. Similarly, at the end of the elastic (linear) region, there is clear evidence for a stick-slip event in all the three structures with different thicknesses. Furthermore, the plastic region, while not strickly linear, requires a very short time to reach its steady state, which is reminescent of a brittle material. As the sliding velocity increases, however, a notable change takes place. While there is a clear transition at low sliding velocities that represents the stick-slip motion, at higher velocities the transition is less evident. Indeed, for $V=2$ m/s there is a larger difference in the maximum shear stress of the three systems, with Structure C exhibiting the lowest value, and the time for the material to reach steady shear stress–shear strain relationship being longer than those with the lower velocity.

**Figure 6:**
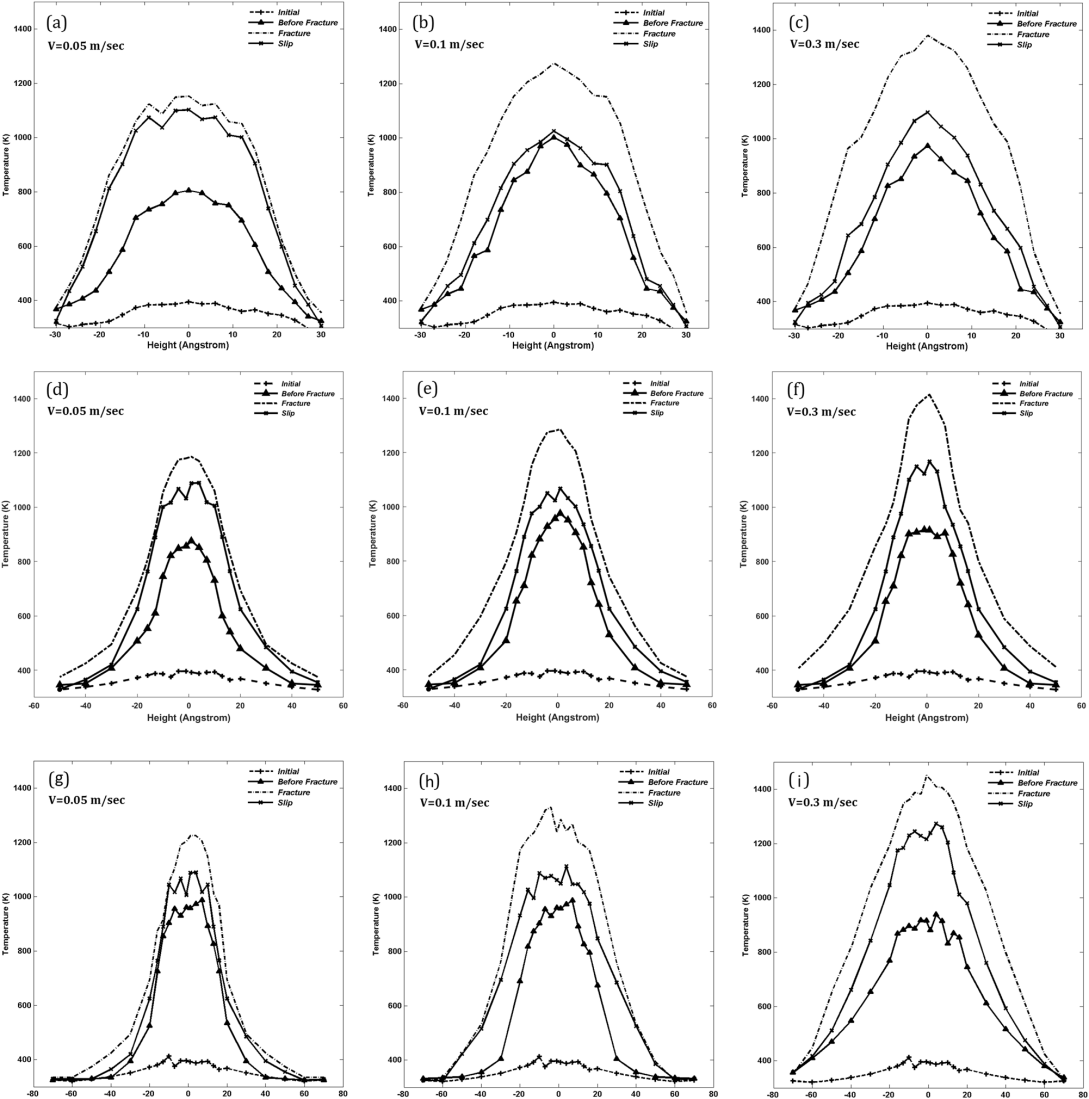
Temperature profile across the amorphous region in the three Structure, A, B, and C, immediately before, at, and after flash heating events for several velocities, and their comparison with the initial profiles. (**a**–**c**) In structure A; (**d**–**f**) in Structure B, and (**g**–**i**) in Structure C.

Figure 6 presents the temperature profiles at the interface at four points in the three structures during the development of flash heating. The profile denoted by “initial” represents the beginning of the simulation, acting as a reference. The next profile represents the interfacial temperature distribution immediately before the aforementioned fracture event, indicating a significant rise in the temperature and approach to flash heating event. The profile referred to as “fracture” represents the distribution *at* flash heating. The profile labeled “slip” is the temperature at the interface *after* the fracture event, and is a measure of the amount of energy that propogates from the interface. There is a significant decrease in the interfacial temperature beyond the flash point in the material with the smallest thickness, indicating a high rate of heat transfer away from the interface. For the material with the largest thickness, on the other hand, the temperature profile beyond the flash, the aforementioned slip profile, does not decrease as much, hence indicating a decreased rate of heat transfer away from the interface.

**Figure 7:**
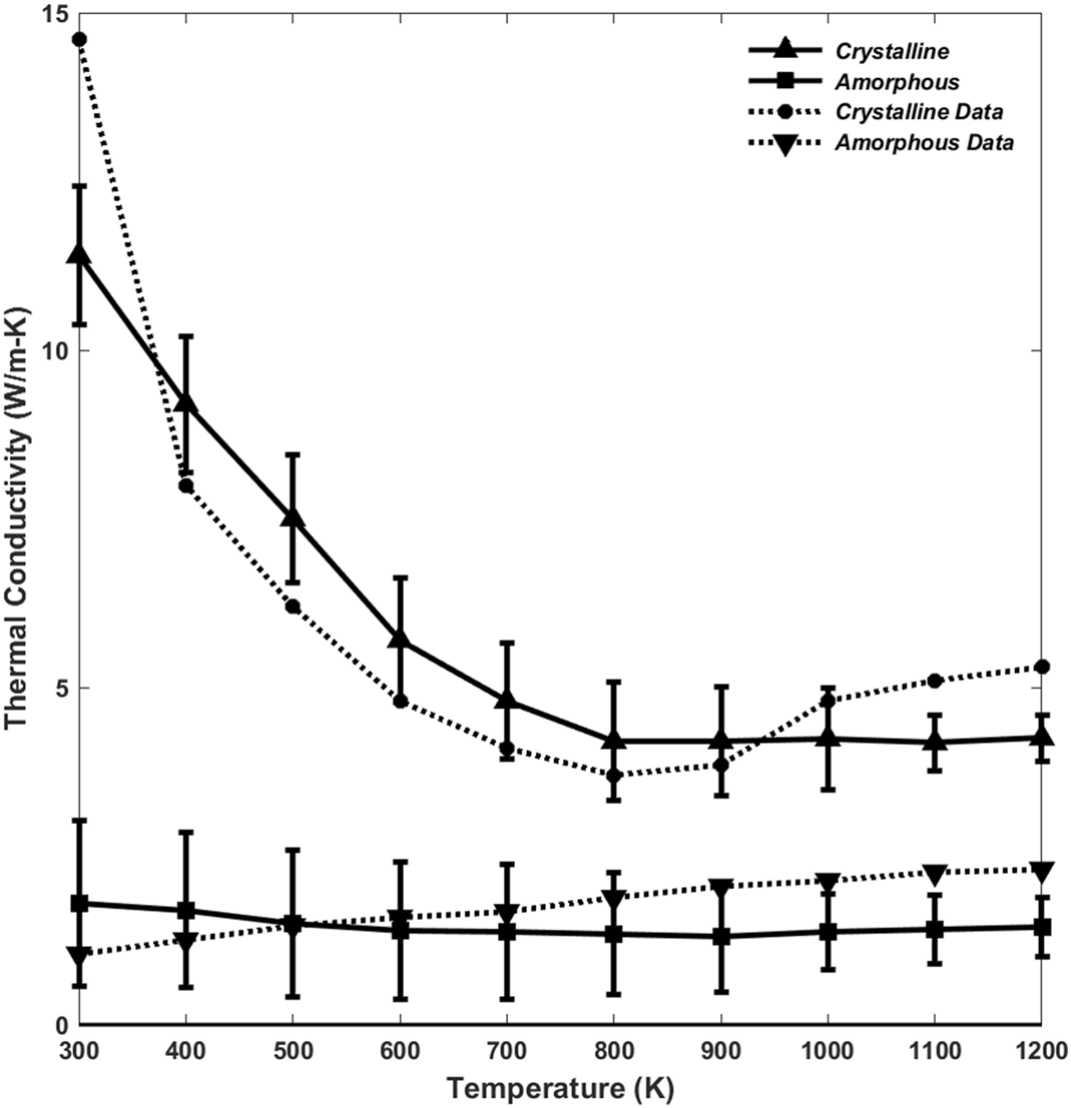
Temperature-dependence of thermal conductivities of amorphous and crystalline quartz and their dependence on temperature. The data for crystalline quartz are from Ref.^26^, while those for amorphous quartz were taken from Ref.^27^.

The underlying mechanism of this phenomenon is the formation of an amorphous layer, formed as a result of flash heating events, which results in the development of a thermally-induced region with reduced strength. To provide further evidence for this, we used MD simulation to compute thermal conductivity of both amorphous and crystalline quartz and its dependence on temperature. The results are shown in Fig. 7, where we also compare them with those of crystalline quartz computed by more limited MD simulations^26^ and with the experimental data for amorphous quartz^27^. The error bars are due to averaging over 15 realizations of the system. The conductivity of amorphous quartz is significantly smaller than that of the crystalline one. Thus, as the thickness of the amorphous region increases, heat transfer from it slows down significantly. Since in the thicker material the extent of the amorphous region is also larger than that in thinner ones, the temperature at the interface increases sharply, leading to flash heating. Thus, the reduction in the strength of the material, as evidenced by Fig. 5, stems from fatigue as a result of both thermal and mechanical effects.

**Figure 8:**
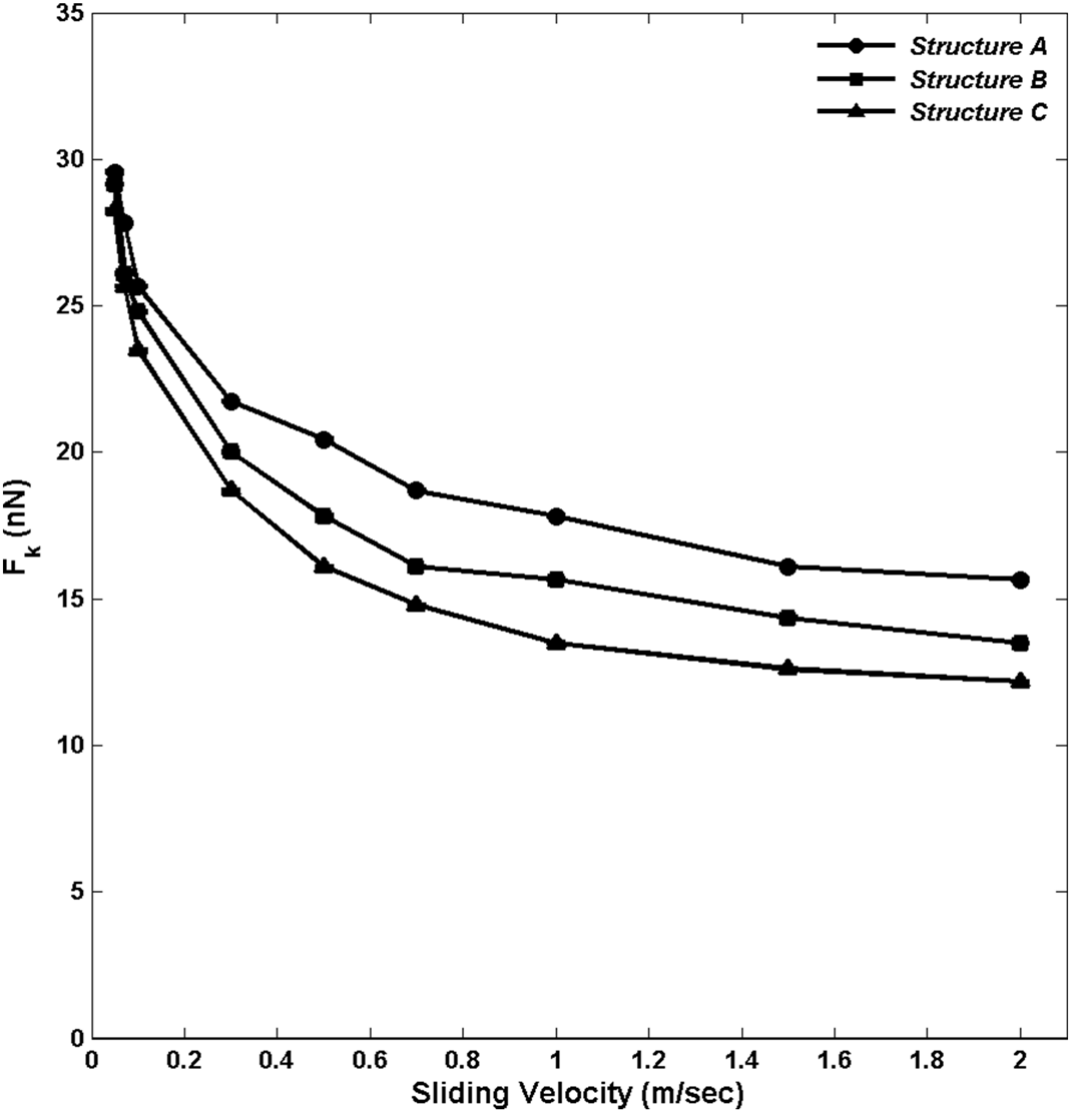
Dependence of the kinetic friction force $F_k$ on the sliding velocity *V*.

Next, we computed the friction force for the top slab using the same method that we used to determine the shearing force for the TL. Figure 8 presents the results for the time-averaged kinetic friction force $F_k$ for the three structures and its dependence on the sliding velocity. There is an initial sharp decay in $F_k$, followed by a behavior that follows roughly, $F_k\propto \ln V$. This is agreement with the experimental work of Goldsby and Tullis^13,15^ on the effect of flash heating on sliding resistance in rock faults. They too reported a qualitatively similar dependence of $F_k$ on the sliding velocity.

Summarizing, this paper studied at molecular scale the root cause of the decay of the interfacial friction coefficient, observed in experiemntal studies of rock and other materials, and attributed to flash heating. As the model of rock material we simulated quartz. The frequency of flash heating events increases with increasing sliding velocity, leaving increasingly shorter times for the material to relax, hence contributing to the increase in the interfacial temperature. If the material is thin, heat is diffused away from the interface region relatively efficiently, which manifests itself in a sharp decrease in the temperature immediately after the flash heating event. But, due to the low conductivity of the amorphous region near the interface, the efficiency of the heat transfer is reduced significantly with increasing thickness, keeping the heat around the interface and producing a weakened material. The weakening behavior is manifested in the stress–strain diagrams, which indicate clearly the decay in in stress, as well as a transition from a brittlelike to ductile behavior as the sliding velocity increases.

## Methods

### The force field and molecular models

The force field (FF) that describes the interactions between the two SiO$_2$ (quartz) surfaces should accurately account for the mechanical properties of quartz, and should also be computationally efficient in order to allow for running of the simulations over long enough time scales. To this end, after testing various FFs, we used one proposed by Tersoff^28,29^, which explicitly accounts for the O–O, O–Si, and Si–Si bonds. All of the FF’s parameters are given in the original references^28,29^, and need not be repeated here.

The unit cell for the crystalline $\alpha$-SiO$_2$ slabs used in the simulations were nonperiodic. As pointed out earlier, three atomistic systems of different thicknesses were generated, each of which contained a pair of equal-size slabs. The thinnest slab, Structure A, consisted of 10,800 atoms, equally divided between Si and O. The second slab, Structure B, was built with 27,000 atoms, while the thickest slab, Structure C, contained a total of 43,200 atoms. In each case the two slabs were identical and, thus, the systems consisted of 21,600, 54,000, and 86,400 atoms for the small, medium and large structures, respectively. All the slabs had the same cross-sectional area. The thickness of the material is important to ascertaining any stabilizing or destabilizing influence that it may have on the interface and heat transfer from it. Clearly, the rate of heat propagation through the material is also influenced by its thickness. Understanding how this manifests itself is one purpose of this study.

The first step in the MD simulation was to ensure that the energy of each individual slab was minimized. Following this step, the total energy of the systems consisting of the two commensurate surfaces were minimized using energy minimization, followed by thermalization by MD simulations. In all the cases the opposing slabs were in direct contact by which we mean that the separation distance between them was small enough to allow for bonding between opposing Si and O atoms at the interface. Afte thermalization of the systems in the *NVT* ensemble, their temperature *T* was raised to 300 K, and then equilibrated in the *NVE* ensemble. The process was performed in a step-by-step manner, with the SiO$_2$ bilayer thermalized by increments of 50 K and equilibrated after each incremental temperature increase. The total time of the subsequent thermalization and equilibration was 7200 ps.

**Figure 9:**
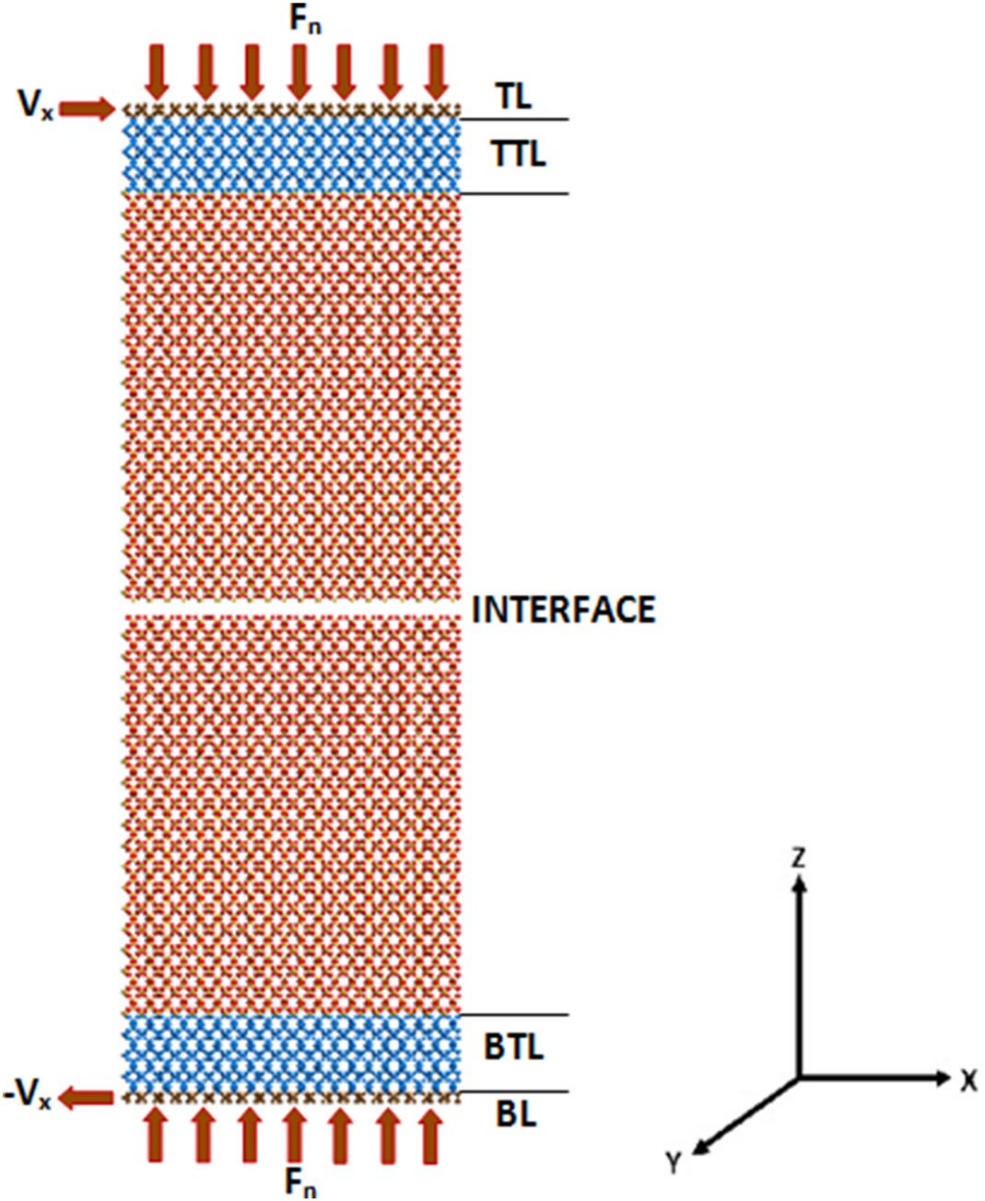
Schematic of the system consisting of two slabs with the top layer (TL), top thermostatted layer (TTL), the interface between the two slabs, the bottom thermostatted layer (BTL), and the bottom layer (BL). $F_n$ is the normal force applied, while $V_x$ is the sliding velocity that pulls the top layer in the *y* direction relative to the bottom slab.

**Figure 10:**
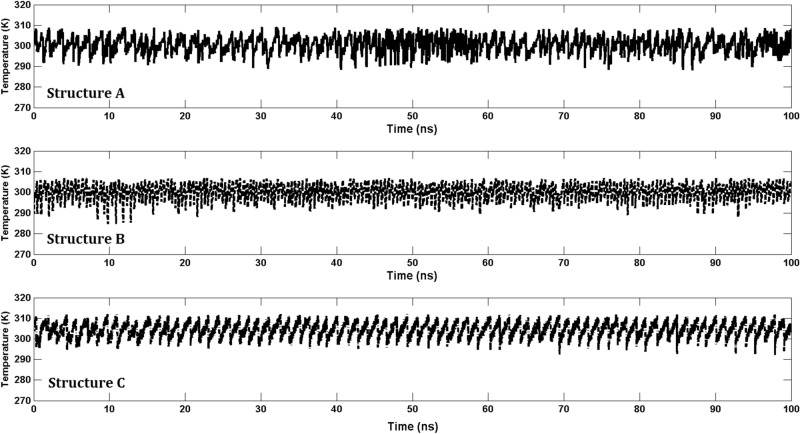
Fluctuation of the temperature in the three structures with various thicknesses.

### Molecular dynamics simulation of sliding friction

Figure 9 shows the details of the molecular system and the anchors to the crystalline bilayer. The simulations were performed in the *NVE* ensemble using the equilibrated interface at 300 K. Sliding friction across the interface was generated through the application of a constant velocity *V*, in order to force the top slab to slide over the bottom one, which allowed determination of the friction force for any given *V*. To properly simulate sliding friction at the interface, the slabs were divided into a series of layers^9,30–32^, which are referred to as the top layer (TL), the top thermostatted layer (TTL), the bottom layer (BL), and the bottom thermostatted layer (BTL). The TTL and BTL were used in order to ensure that the system as a whole remains at a constant temperature of 300 K throughout the course of the MD simulations. The function of the TTL and BTL, as the “heat sinks”, was ensuring that the heat generated at the interface can be attributed solely to the sliding friction. This was achieved by rescaling the velocities of the atoms in the two layers at every time step.

In order to ensure that there is symmetry throughout the system, the TL and BL were subjected to two equal and opposite sets of events. First, to ensure constant loading, the atoms in the TL were subjected to a force in the $-z$ direction, while those in the BL were subjected to an equal force in the $+z$ direction. The resulting sum of the forces was carefully calculated to obtain a net, constant pressure of 1 GPa. For the second event, the atoms in both the TL and BL were held constant at a prescribed velocity. A constant velocity was applied in the $+x$ direction to all the atoms in the TL, while the same velocity was applied in the $-x$ direction to all the atoms in the BL. The simulated velocities were in the range 0.05–2 m/s, well within the range of experiments^13,15^. Due to the wide range of the velocities that we simulated and the fact that we wish to simulate subseismic velocities, very long simulations were carried out for 100–600 ns. The melting temperature of $\alpha$-SiO$_2$ is about 1700 $^\circ$C. The time step in all the cases was $10^{-3}$ ps.

The sliding distance of the two slabs is denoted by *X*, defined as the difference between the *x* positions of the centers of mass of the two slabs. For all the sliding velocities simulated, the temperature in the thermostatted layers—the TTL and BTL—did not fluctuate by any significant amount, as they did not exceed by more than 10 percent of the average temperature, $T\approx 300$ K. Figure 10 presents the temperature fluctuations in the three structures at a velocity of 0.3 m/s. Similar behavior was observed for all other sliding velocities.
